# Structural determinants of human ζ-globin mRNA stability

**DOI:** 10.1186/1756-8722-7-35

**Published:** 2014-04-21

**Authors:** Zhenning He, Decheng Song, Sebastiaan van Zalen, J Eric Russell

**Affiliations:** 1 Department of Medicine (Hematology/Oncology), Perelman School of Medicine at the University of Pennsylvania, Biomedical Research Building, Room 808, 421 Curie Boulevard, Philadelphia, PA 19104, USA; 2 Department of Pediatrics (Hematology), Perelman School of Medicine at the University of Pennsylvania, Philadelphia, PA, USA

**Keywords:** RNA stability, ζ Globin, Sickle-cell disease, Thalassemia

## Abstract

**Background:**

The normal accumulation of adult α and β globins in definitive erythrocytes is critically dependent upon processes that ensure that the cognate mRNAs are maintained at high levels in transcriptionally silent, but translationally active progenitor cells. The impact of these post-transcriptional regulatory events on the expression of embryonic ζ globin is not known, as its encoding mRNA is not normally transcribed during adult erythropoiesis. Recently, though, ζ globin has been recognized as a potential therapeutic for α thalassemia and sickle-cell disease, raising practical questions about constitutive post-transcriptional processes that may enhance, or possibly prohibit, the expression of exogenous or derepresssed endogenous ζ-globin genes in definitive erythroid progenitors.

**Methods:**

The present study assesses mRNA half-life in intact cells using a pulse-chase approach; identifies *cis*-acting determinants of ζ-globin mRNA stability using a saturation mutagenesis strategy; establishes critical 3′UTR secondary structures using an *in vitro* enzymatic mapping method; and identifies *trans*-acting effector factors using an affinity chromatographical procedure.

**Results:**

We specify a tetranucleotide 3′UTR motif that is required for the high-level accumulation of ζ-globin transcripts in cultured cells, and formally demonstrate that it prolongs their cytoplasmic half-lives. Surprisingly, the ζ-globin mRNA stability motif does not function autonomously, predicting an activity that is subject to structural constraints that we subsequently specify. Additional studies demonstrate that the ζ-globin mRNA stability motif is targeted by AUF1, a ubiquitous RNA-binding protein that enhances the half-life of adult β-globin mRNA, suggesting commonalities in post-transcriptional processes that regulate globin transcripts at all stages of mammalian development.

**Conclusions:**

These data demonstrate a mechanism for ζ-globin mRNA stability that exists in definitive erythropoiesis and is available for therapeutic manipulation in α thalassemia and sickle-cell disease.

## Background

Human α-like globins are encoded by three homologous genes (5′-ζ-α2-α1-3′) arranged in order of their developmental expression [1]. Embryonic α-like ζ globin is produced during the first gestational trimester in primitive erythroblasts that originate in blood islands of the yolk sac, while fetal/adult α globin is induced at the end of this interval and continues to express in definitive erythroblasts that are initially produced in the liver and, subsequently, in the bone marrow [1,2]. Unlike α globin--which is required for normal growth and development both in the uterus and after birth--ζ globin appears to be largely dispensable to mammalian reproduction. Embryonic ζ globin is not required for viability in mice [3], and naturally occurring deletions and duplications in its encoding gene are not associated with any defined phenotype in man [2,4,5]. As a consequence of this apparent biological irrelevance, then, there has been little incentive to detail the processes that control ζ-globin expression in either primitive or definitive erythropoiesis.

Investigations of the molecular and cellular programs that regulate ζ globin, though, are recently justified by its demonstrated potential as a novel therapeutic for both α thalassemia and sickle-cell disease [6,7]. When compelled to express in definitive erythroid cells, embryonic ζ globin assembles with adult β globin into heterotetrameric Hb ζ_2_β_2_ (Hb Portland-2), which exhibits O_2_-binding and allosteric properties that differ modestly from those of Hb α_2_β_2_ (Hb A, the principal adult hemoglobin), but that remain fully compatible with normal adult physiology [8,9]. The significance of this approach for treating α-globin deficiency states (α thalassemia) is illustrated by evidence that transgenic human ζ globin fully reverts the pathological phenotype of mice containing heterozygous knockout of their endogenous α-globin genes and, remarkably, restores full viability to animals with homozygous, embryonic-lethal inactivation of these same genes [6]. The developmental stage-discordant expression of ζ globin holds additional therapeutic promise for sickle-cell disease, as its low-level expression effectively mitigates the abnormal phenotype of mouse models of this disorder [7,10]. Importantly, the α → ζ exchange that converts pathological Hb α_2_β^S^_2_ (Hb S) to non-pathological Hb ζ_2_β^S^_2_ does not exclude the mutant β^S^ subunit, raising the possibility that this novel strategy can be coordinated with contemporary therapies that promote β^S^ → γ exchange. These two developing applications for ζ globin--effected through either gene-reactivation or gene-supplementation strategies--now recommend careful description of the events that contribute to its regulation in definitive erythroid cells.

Post-transcriptional regulatory processes are critical to the normal accumulation of adult α- and β-globins in definitive erythrocytes, ensuring that the cognate mRNAs are maintained at high levels in transcriptionally silent--but translationally active--progenitor cells. The fundamental bases for the prolonged half-life (t_1/2_) values for both α- and β-globin mRNAs have now been established in detail, including the identification of specific *trans*-acting effector proteins and the *cis*-acting regulatory elements that they target [11-16]. Similar analyses of embryonic ζ-globin transcripts that are expressed either physiologically or following therapeutic derepression in definitive erythropoiesis indicate that the mRNAs are subject to post-transcriptional processes that are constitutive to these cells [17,18]. While ζ-globin mRNA is observed at low levels in normal adult erythroid progenitors [19-21], it can accumulate to significantly higher levels in cells where its encoding gene is transcriptionally dysregulated [22-24], implicating the presence of active post-transcriptional processes that are capable of acting upon developmental stage-discordant ζ-globin mRNA. Transgenic human ζ globin can also be produced in abundance in adult mice (at levels that support viability in α-globin nullizygotes) [6], validating the assertion that--as a practical matter--the half-life of its encoding mRNA is sufficiently long to ensure that biologically relevant levels of ζ globin are translated [6-9]. These principles are confirmed in individuals with Southeast Asian-type α thalassemia [25], congenital dyserythropoietic anemia [26], and juvenile chronic myelocytic leukemia [24], who express measurable quantities of both ζ-globin mRNA and ζ-globin protein. Collectively, these observations endorse the likelihood that post-transcriptional programs in adult erythropoiesis are fully permissive for the expression of ζ-globin mRNA.

The present work describes structures within the ζ-globin 3′UTR that are critical to its post-transcriptional regulation, illustrates their effects on the half-life value of the full-length ζ-globin mRNA in intact cells, and identifies a *trans*-acting factor that is likely to mediate this process. These results provide a foundation for understanding the impact of post-transcriptional activities to the developmental stage-discordant expression of ζ globin in adult erythropoiesis, as a promising therapeutic for both α thalassemia and sickle-cell disease.

## Results

### A transcriptional chase strategy identifies *cis*-acting regulatory determinants within the ζ-globin 3′UTR

**Figure 1:**
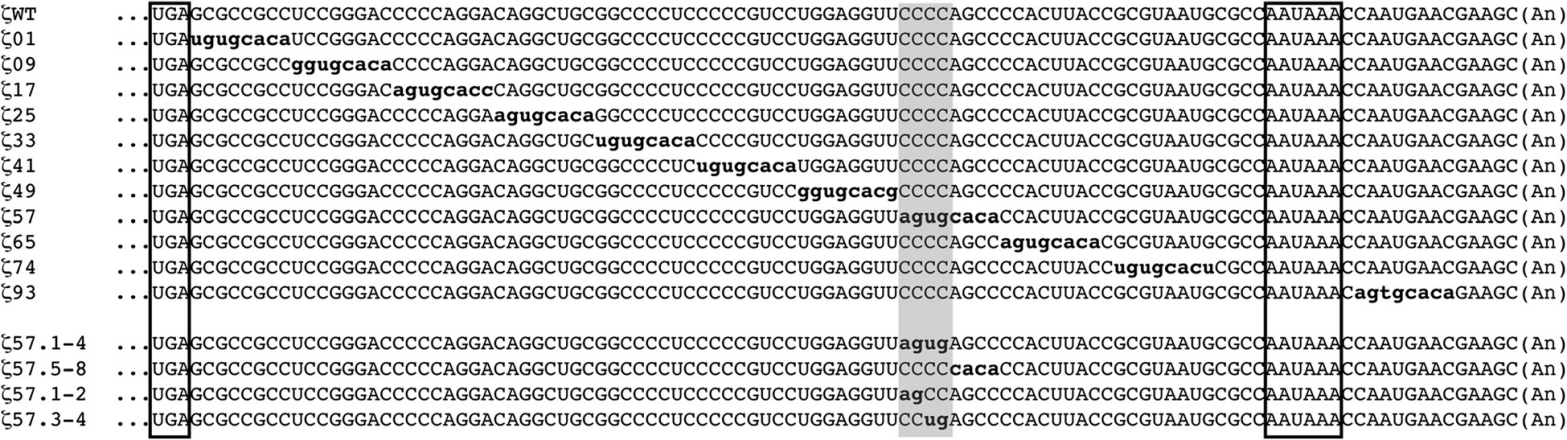
**Primary structures of mRNAs encoded by wild-type and 3′UTR-variant ζ-globin mRNAs.** Native and mutated regions of each 3′UTR sequence are denoted in uppercase and lowercase bold, respectively. The UGA termination codon and AAUAAA polyadenylation signal are boxed; the polyadenylate tail is indicated as (A_n_). The tetranucleotide motif that confers stability to the ζWT mRNA is highlighted in gray.

Transgenic human ζ globin accumulates to high levels in mouse erythrocytes, consistent with the existence of *cis*-acting determinants that stabilize the cognate ζ-globin mRNA in transcriptionally silent progenitor cells [6,7,18]. Precedent analyses of both globin and non-globin mRNAs indicate that relevant stability determinants are commonly confined to the 3′ region of untranslated mRNA (3′UTR), which is not subject to disruption by translating ribosomes [27-31]. We investigated the likely positioning of an mRNA stability element in the ζ-globin 3′UTR in a systematic manner, employing a linker-scanning strategy to identify *cis*-acting determinants of mRNA half-life that act *in vivo* in intact cells. Full-length ζ-globin genes were constructed to include either the 105-nt wild-type ζ-globin 3′UTR (ζWT); or identically sized variant ζ-globin 3′UTRs, each containing an 8-nt mutation at a different position between the UGA translational termination codon and the polyadenylate tail (Figure 1). Each gene was linked to a recombinant hybrid tetracycline response element (TRE) that promotes transcription in cultured cells expressing a hybrid tetracycline *trans*-activating protein (tTA), but is transcriptionally silent in the presence of tetracycline (tet) [32]. This approach permitted half-life values for wild-type and 3′UTR-variant ζ-globin mRNAs to be specified *in vivo* in tTA-expressing cells, using a transcriptional chase strategy that assesses the temporal reduction in the level of each variant ζ-globin mRNA relative to the steady-state level of a tet-indifferent control mRNA [33,34].

**Figure 2:**
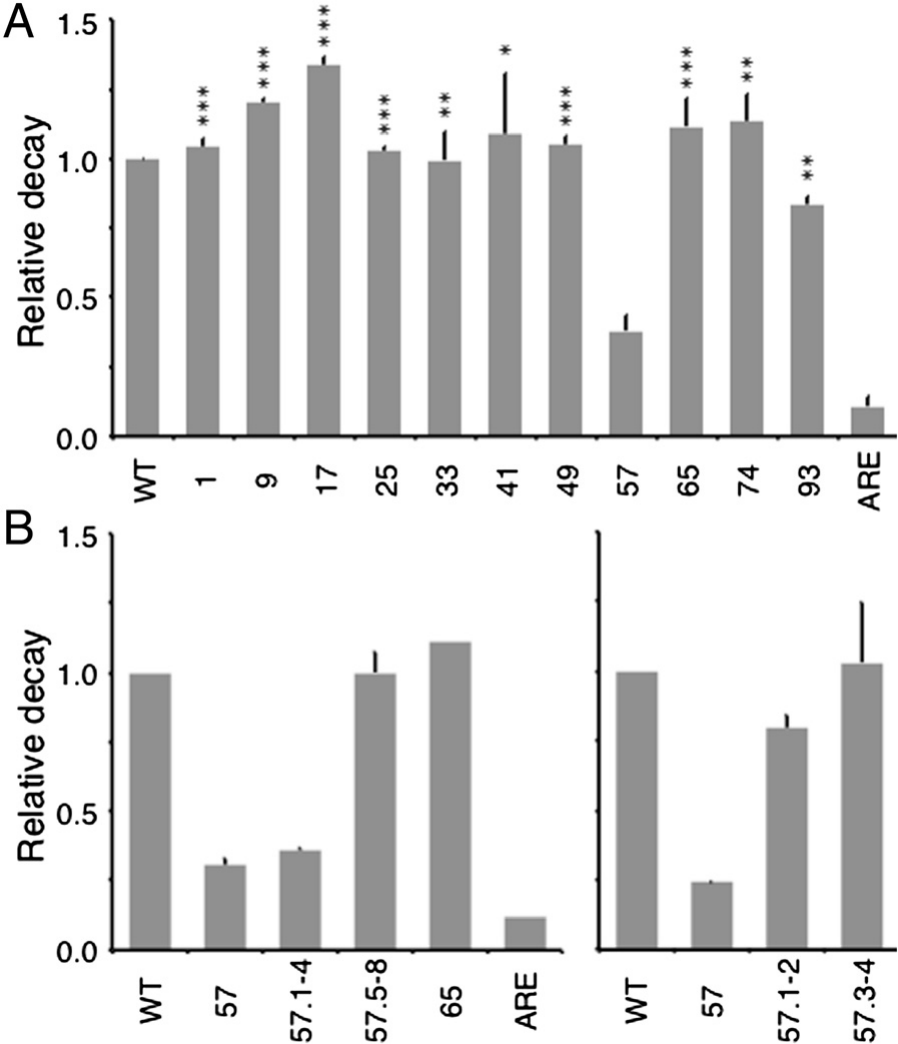
**Relative decay of transiently expressed wild-type and 3′UTR-variant ζ-globin mRNAs *in vivo*.** Values correspond to the quantity of each ζ-globin mRNA remaining after a 16-hr transcriptional chase interval, relative to transiently co-expressed β-globin mRNA. The relative decay of ζWT mRNA was arbitrarily assigned unit value. Each bar represents the mean ± SD value from between two and six replicates. The relative decay of ζARE mRNA, which contains an mRNA-destabilizing element within its 3′UTR [33,37], was assessed in all experiments as a methodological control. **(A)** Relative decay of 3′UTR-variant ζ-globin mRNAs carrying octanucleotide mutations. Asterisks indicate p values for each mRNA, relative to ζ57 mRNA (* < 0.02; ** < 0.005; *** < 0.001). **(B)** Relative decay of 3′UTR-variant ζ-globin mRNAs carrying tetranucleotide mutations in the initial (ζ57.1-4) or terminal (ζ57.5-8) four nucleotides of the nt 57–64 motif (*left*), or smaller mutations in the first (ζ57.1-2) or second (ζ57.3-4) dinucleotide of this region.

To identify the positions of mRNA stability determinants within the ζ-globin 3′UTR, we conducted a screening assay that quantified the relative decay of wild-type and 3′UTR-variant ζ-globin mRNAs *in vivo* in intact cultures. Mammalian cultured-cell models for erythroid development transcribe a variety of embryonic, fetal, and adult globin mRNAs that can compete for post-transcriptional regulatory activities [18,35,36]; consequently, we conducted our studies in tTA-expressing HeLa cells (HeLa^tTA^) that do not transcribe globin mRNAs and have previously been used to characterize post-transcriptional processes affecting both native and exogenous globin genes [16,34]. We tested the relative decays of transiently expressed ζWT and 3′UTR-variant ζ-globin mRNAs, relative to that of a control β-globin mRNA transcribed from a co-transfected gene, using a two-point decay method of our design. One of the 3′UTR-variant mRNAs--containing a CCCCAGCC → agtgcaCa substitution at nts 57–64 (ζ57 mRNA)--reproducibly decayed over a 16-hour interval to a level that was approximately one-third that of ζWT mRNA (Figure 2A). The effect of the octanucleotide substitution was dependent upon its position rather than its content, as ζ-globin mRNAs containing a similar mutation elsewhere in the 3′UTR--including flanking nts 49–56 and 65–72 (ζ49 and ζ65 mRNAs, respectively)--decayed at the same rate as parental ζWT mRNA. The mutational effects were consistent over three or more replicate analyses, thus identifying and localizing a previously unknown post-transcriptional determinant of mRNA decay within the ζ-globin 3′UTR. For convenience, we now term the nt 57–64 region the ζ*-globin m*RNA *r*egulatory *e*lement (ZMRE).

We posited that the kinetics of ζ-globin mRNA decay might be similarly affected by a smaller defect in the nt 57–64 target region, according with precedent studies demonstrating the deleterious effects of site-specific single- or several-nt 3′UTR substitutions on the stabilities of both α- and β-globin mRNAs [16,18,38]. This hypothesis was validated by transcriptional chase analyses of transiently expressed ζ-globin mRNAs carrying tetranucleotide substitutions at either positions 1–4 or 5–8 of the ζ57 target region (ζ57.1-4 and ζ57.5-8 mRNAs, respectively; CCCCAGCC **→** agtgAGCC and CCCCAGCC **→** CCCCcaCa). While the relative decay of ζ57.5-8 mRNA was no different from ζWT mRNA (Figure 2B, *left*), the decay of ζ57.1-4 mRNA was significantly accelerated, and to a similar extent as that of parental ζ57 mRNA. Subsequent analyses demonstrated that smaller, dinucleotide substitutions to the nt 57–64 region did not affect mRNA decay, indicating that the ZMRE is functionally permissive for mutations of this magnitude (Figure 2B, *right*). Collectively, these screening data specify the importance of a site-specific tetranucleotide motif to the regulated accumulation of ζ-globin mRNA.

### The ZMRE regulates the half-life of ζ-globin mRNA

**Figure 3:**
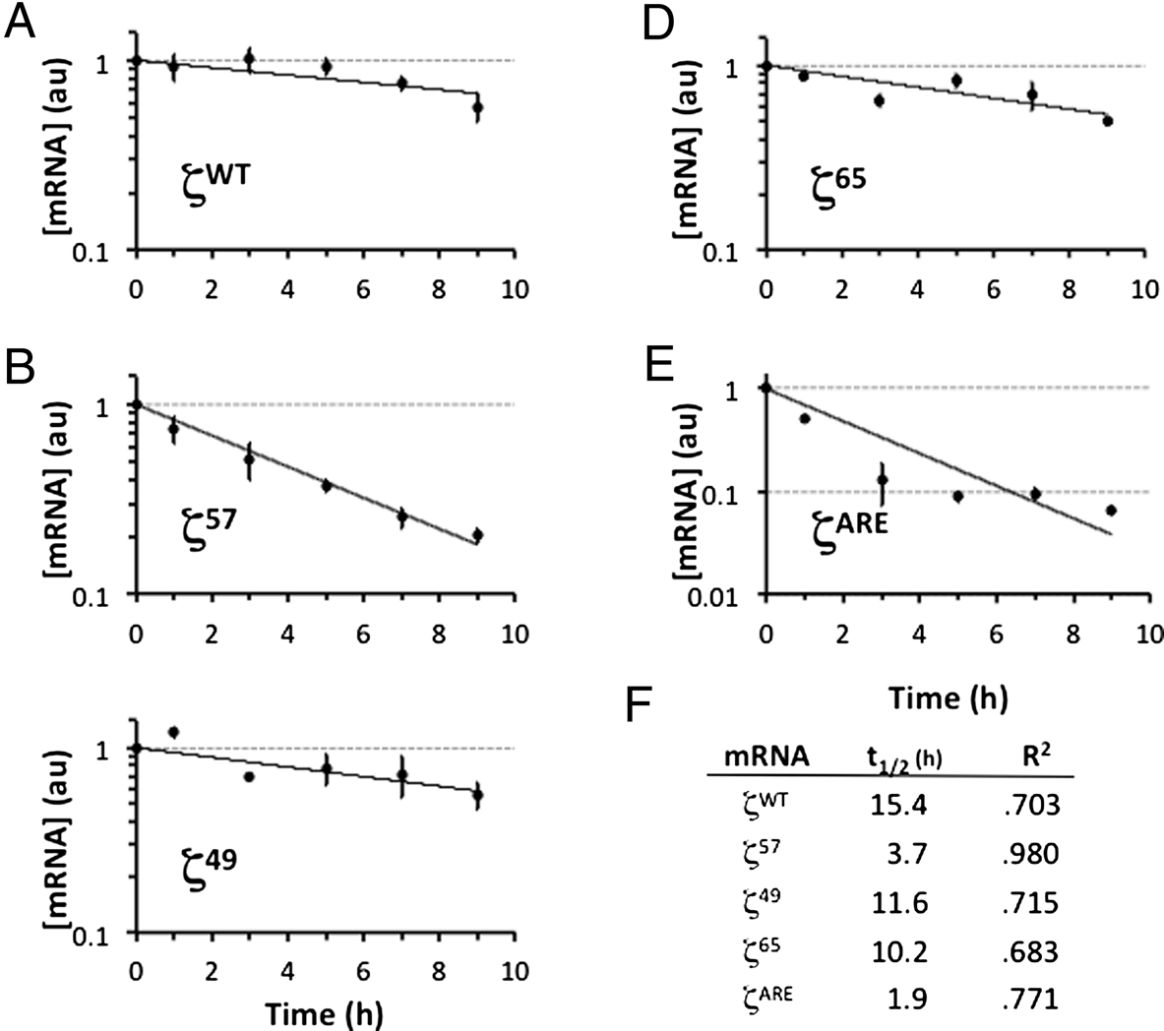
**Half-life values for 3′UTR-variant ζ-globin mRNAs.** Transiently transfected HeLa^tTA^ cells were amended with tet, and levels of ζ-globin mRNA assessed at defined intervals relative to levels of endogenous tet-indifferent β-actin mRNA. The normalized level of ζ-globin mRNA at each time point is plotted relative to the normalized level at t = 0, which is arbitrarily assigned unit value. Each point depicts the mean ± SD value for at least three replicates. **(A)** Stable control ζWT mRNA. **(B)** ζ57 mRNA. **(C)** ζ49 mRNA. **(D)** ζ65 mRNA. **(E)** Unstable control ζARE mRNA. **(F)** Half-life and R^2^ values for curves in panels A-E, calculated from best-fit exponential decay curves with a fixed value of 1.0 at t = 0.

**Figure 4:**
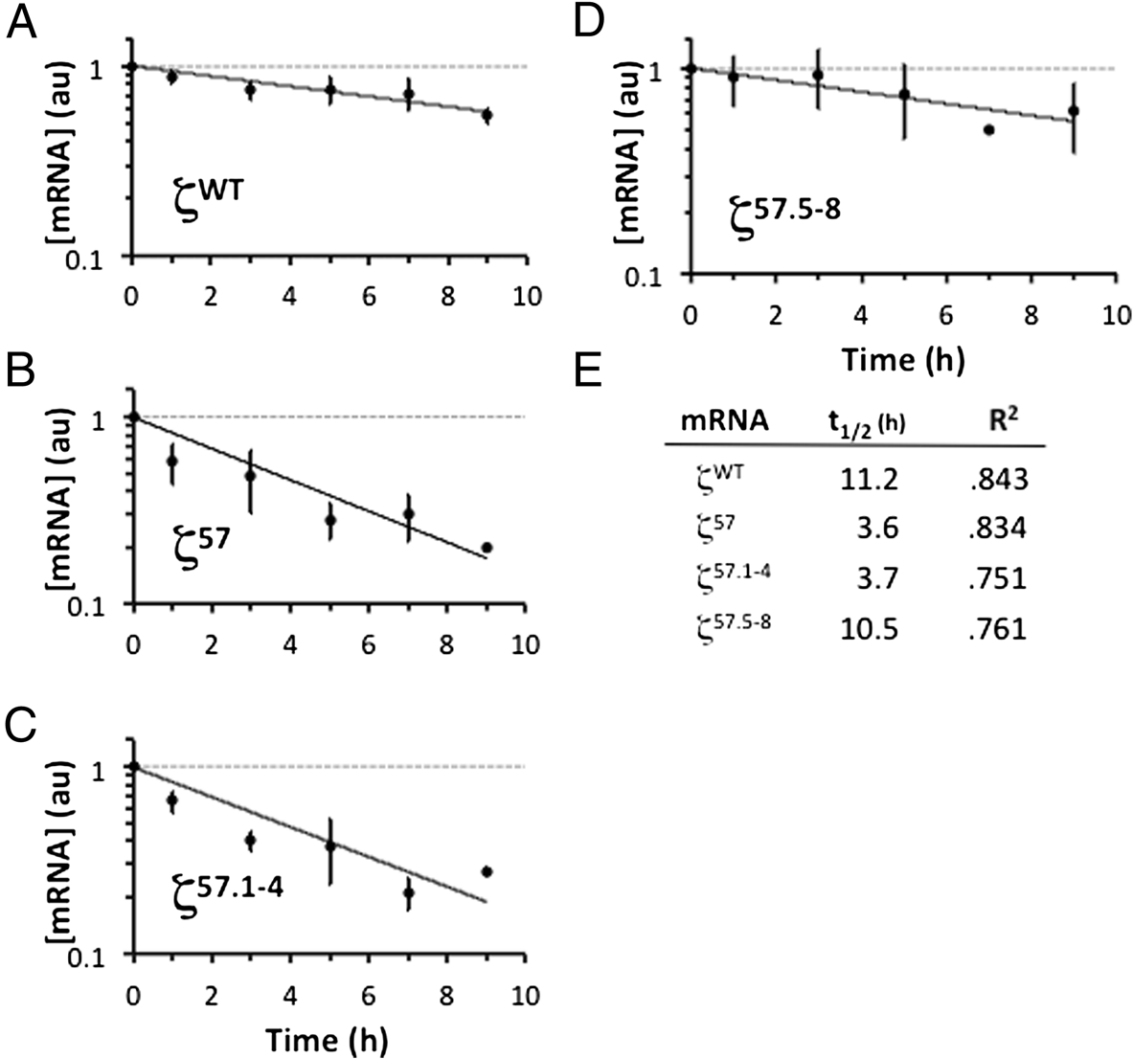
**Half-life values for ζ57-derivative globin mRNAs.** Stability analyses were conducted on informative ζ-globin mRNAs as described in Figure 3. **(A)** Stable control ζWT mRNA. **(B)** ζ57 mRNA. **(C)** ζ57.1-4 mRNA. **(D)** ζ57.5-8 mRNA. **(E)** Half-life and R^2^ values for curves in panels A-D, calculated from best-fit exponential decay curves.

Results from two-point screening analyses indicated the likelihood that the ζ57 determinant is essential for normal accumulation of ζ-globin mRNA in cell cytoplasm. We validated this hypothesis by formally assessing t_1/2_ values for wild-type and informative 3′UTR-variant ζ-globin mRNAs *in vivo*. Analyses were conducted in triplicate in transiently transfected HeLa^tTA^ cells using a conventional pulse-chase strategy that assesses the levels of test mRNAs at defined intervals, relative to the level of endogenous tet-indifferent β-actin mRNA [33]. Under these conditions, the ζ57 mRNA displayed a t_1/2_ value that was approximately one-fourth that of ζWT mRNA (3.7 *v* 15.4 hr, respectively), while ζ49 and ζ65 mRNAs--containing ZMRE-flanking mutations--displayed relatively normal t_1/2_ values of 11.6 and 10.2 hr (Figure 3). These data were affirmed by subsequent analyses demonstrating the deleterious effect the ζ57.1-4 mutation (but not the ζ57.5-8 mutation) on the half-life of ζ-globin mRNA (Figure 4). In these latter studies, the t_1/2_ value of control ζWT mRNA (11.2 hr) was reduced to the same extent by either full (ζ57) or partial (ζ57.1-4) substitution of the nt 57–64 target region (3.6 and 3.7 hr, respectively), confirming the centrality of the CCCC tetranucleotide to ZMRE function. Additional pulse-chase studies--conducted in cells that *stably* express wild-type and 3′UTR-variant ζ-globin mRNAs--revealed similar results (not shown), providing a third measure of experimental validation. Collectively, the transcriptional chase analyses agree on the positioning of a post-transcriptional regulatory element within the ζ57 region, as well on its critical importance to the constitutive half-life of ζ-globin mRNA.

### The mRNA-stabilizing activity of the ZMRE is not autonomous

**Figure 5:**
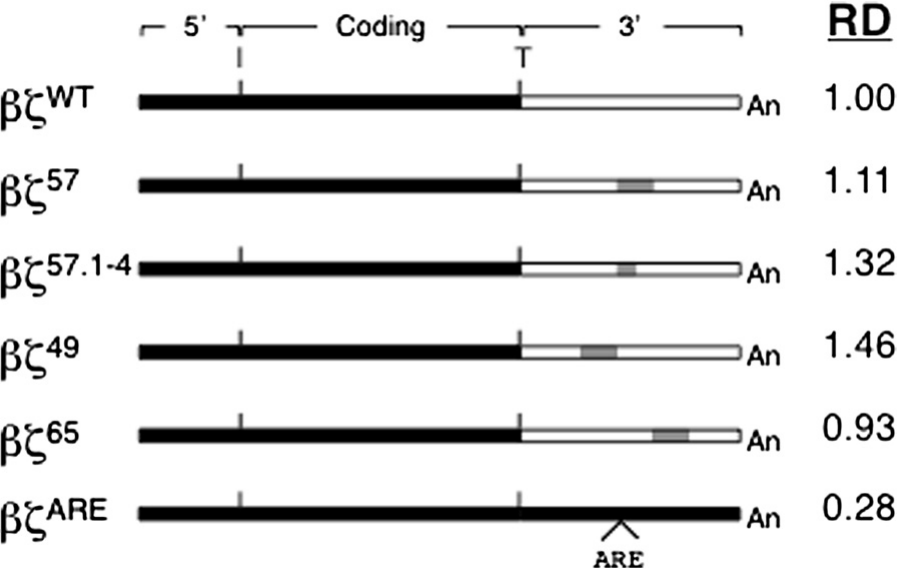
**Relative decay of transiently expressed βζ-chimeric mRNAs in HeLa**^**tTA **^**cells.** (A) Structures of mRNAs encoded by chimeric βζ-globin genes; regions derived from β- and ζ-globin sequence are indicated in black and white, respectively. The positions of the translation initiation (I) and termination (T) sites, as well as the polyadenylate tail (An), are indicated. The ζ57, ζ57.1-4, ζ49, and ζ65 mutations are illustrated in gray. The 3′UTR of the βζARE mRNA contains a defined 59-nt *a*denosine/uridine-*r*ich *e*lement, inserted 15 nts 3′ to the translation stop codon; this motif destabilizes the full-length transcript, providing an unstable mRNA that is used as a methodological control [33]. RD = relative decay values obtained using the experimental strategy described in Figure 3.

Previous analyses in transgenic mice demonstrated that the stability of human α-globin mRNA was significantly reduced when its 3′UTR was exchanged for the corresponding region of ζ-globin mRNA [18]. This effect was originally attributed to a generic difference in then-unspecified determinants of α- and ζ-globin half-life [17] and, later, to sequence dissimilarities between homologous pyrimidine-rich regulatory elements within the α- and ζ-globin 3′UTRs [18]. We posited that this effect might alternately arise from a ZMRE function that was highly sensitive to its regional mRNA structural context; i.e., that the regulatory properties of the ZMRE might be reduced when repositioned near heterologous mRNA. To test this possibility, we assessed whether the half-life of a reporter coding-region mRNA contiguous with the ζ57 3′UTR would be reduced relative to an identical mRNA flanked by a ζWT 3′UTR (Figure 5A). A two-point decay analysis indicated that the stabilities of 3′UTR-chimeric β-globin mRNAs were indifferent to the presence of stabilizing (ζWT) or destabilizing (ζ57 and ζ57.1-4) 3′UTRs, validating an activity for the ZMRE that is dependent upon its RNA context (Figure 5B). While it is difficult to generalize this effect to coding regions from all mRNAs, our observations suggest that the activity of the ZMRE is defined both by its primary structure (Figures 3 and 4) as well as by undefined effects of neighboring mRNA and/or mRNA-bound factors (Figure 5).

### The ZMRE maps to a region of ζ-globin 3′UTR that displays conformational flexibility

**Figure 6:**
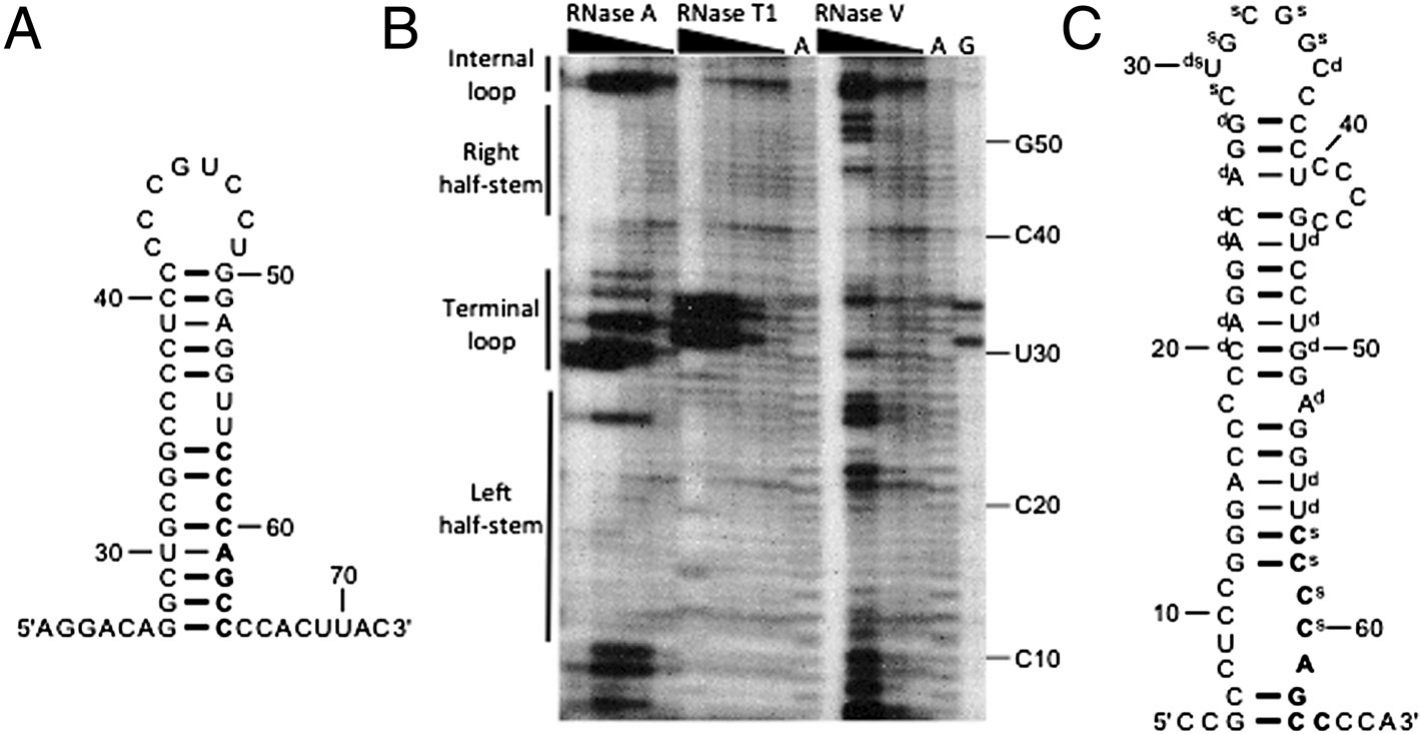
**High-order structure of the ζ-globin 3′UTR. (A)** A thermodynamically favored structure resulting from unrefined m-fold analysis of the full-length ζ-globin 3′UTR (detail). The ZMRE is indicated in boldface. Double and triple hydrogen bond interactions are indicated by thin and thick connectors, respectively. **(B)** Enzymatic secondary-structure mapping of the ζ-globin 3′UTR (detail). 5′-end [^32^P]-labeled RNAs corresponding to the ζ-globin 3′UTR (and contiguous 18-nt polyadenylate tail) were digested with RNases A, T1, and V1 at four different concentrations (wedges), then resolved on an acrylamide-urea gel. Nucleotide assignments (right) were deduced by aligning the known 3′UTR sequence to specific bands corresponding to guanine bases (lane G), which were generated by RNase T1 digestion of denatured 3′UTRs. An alkaline hydrolysis ladder (lanes **A)** provides additional sequence information at single-nucleotide resolution. Regions of 3′UTR that exhibit stem- and loop-like characteristics (i.e., sensitivities to double- and single-strand-specific nucleases, respectively) are indicated to the left. **(C)** Thermodynamically favored structure resulting from m-fold analysis of the full-length ζ-globin 3′UTR, where base-pairing is enforced for nts 20–21, 24–26, and 38; and prohibited for nts 29–34 (detail). Nucleotides that displayed less-exacting single- or double-strand characteristics were not used for predictive purposes but are indicated by s and d, respectively.

The observation that the ζ-globin mRNA is destabilized by mutations within the ZMRE, but not by similar mutations in neighboring cytosine-rich regions, suggests a local high-order structure that favors functional interactions between the ZMRE and one or more *trans*-acting effector factors. An unrefined m-fold analysis of ζ-globin 3′UTR structure predicts that the ZMRE is embedded in a highly stable RNA stem, and directly participates in seven base-pair interactions (Figure 6A) [39,40]. We were skeptical that this arrangement would support site-specific interactions with regulatory RNA-binding proteins, and consequently employed an enzymatic mapping technique to formally characterize the high-order RNA structure that encompasses the ZMRE [41]. Our analyses revealed regions of ζ-globin 3′UTR that--by virtue of their sensitivities to single- and double-strand-specific RNases--were inconsistent with the unrefined structure (Figure 6B). A subsequent m-fold analysis, constrained by unequivocal, experimentally determined regions of single- and double-stranded interactions, predicted an alternate structure with a favorable ΔG^o^ value (−26.3 kcal/mol) in which the nt 57–64 target region is positioned on an asymmetrical interior loop, contiguous to a 16-nt stem (Figure 6C). This arrangement--which would be expected to facilitate conformational flexibility in the regions of RNA that flank the ZMRE [42]--would also account for our observation that transitional nucleotides (bordering the base-paired and unpaired regions) are assigned double-stranded interactions but display modest sensitivities to single-strand nucleases (e.g., C57 and C58). The resistance of the cytosine-rich ZMRE to double-strand-specific RNase V may additionally suggest potential hydrophobic ‘stacking’ interactions that can serve as a target for *trans*-acting regulatory factors [43]. The structural dynamics of this region, combined with experimental evidence favoring its assembly, indicates that the positioning of the ZMRE may be critical to its regulatory function.

### Cytoplasmic mRNA-binding proteins interact with the ZMRE

**Figure 7:**
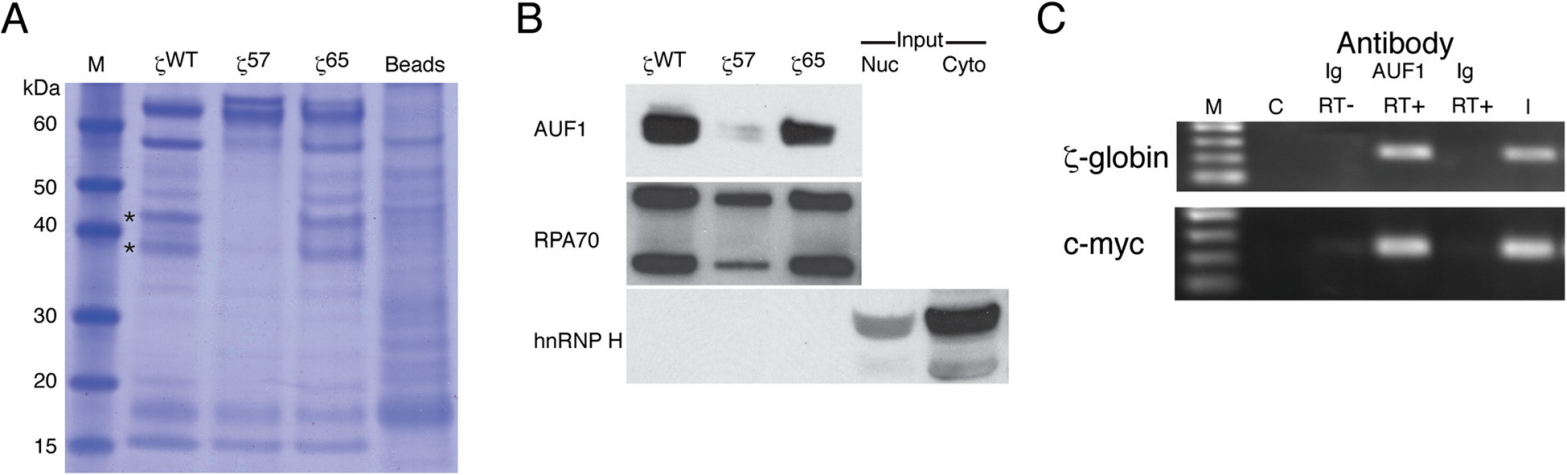
**Affinity-enrichment analyses for ZMRE-binding factors. (A)** SDS-PAGE of retentate from ζWT, ζ57, and ζ65 ssDNA probes, and from control agarose beads. Bands corresponding to two AUF1 isotypes are indicated with an asterisk. M = molecular size marker. **(B)** Confirmatory immunoblot analyses. Retentate was assessed using antibodies against AUF1, RPA70 (positive control, binds all three probes), and hnRNP H (negative control, present in input nuclear and cytoplasmic fractions). Similar results were obtained in parallel analyses of extracts from HeLa cells (not shown). **(C)** RNA immunoprecipitation analyses. Experiments were conducted on extract from HeLa cells that express ζ-globin mRNA, using AUF1 antibody or control polyspecific immunoglobulin (Ig) control. c-Myc mRNA was assessed in parallel as a positive control for AUF1 binding. M = molecular size marker; C = water-only RT-PCR control; I = control input (unprecipitated) mRNA.

The stabilities of many mRNAs--including those encoding α globin [27], β globin [16], α(I) collagen [28], tyrosine hydroxylase [29], histone [30], and the transferrin receptor [31]--require the assembly of defined mRNP effector complexes on specific determinants within their 3′UTRs. To identify candidate *trans*-acting factors that might functionally interact with the ZMRE, we conducted affinity chromatography analyses on cell extracts from both non-erythroid HeLa and erythroid K562 cells, using three 32-nt single-stranded (ss) DNAs corresponding to comparable regions of ζ57, control ζWT, and control ζ65 3′UTRs. Both the control ζWT and ζ65 ssDNAs retained fewer than 10 distinct factors--two of which were not retained by the ζ57 ssDNA--suggesting site-specific *trans*-acting interactions and potential regulatory functions (Figure 7A). Excised bands were subjected to nanoLC/MS/MS and provisionally identified as isoforms of AUF1 (hnRNP D), a protein that is ubiquitously expressed [44], displays known mRNA-binding characteristics [45,46], participates in post-transcriptional regulatory events that effect both mRNA stabilization [47] and destabilization [48], and has been implicated as a determinant of β-globin mRNA half-life [16]. The identity of AUF1--and its relative affinities for ζWT, ζ57, and specificity-control ζ65 3′UTRs--was subsequently validated by immunoblot analyses of the three retentates (Figure 7B). Additional analyses demonstrated that AUF1 immunoprecipitate of extract prepared from ζWT-expressing HeLa^tTA^ cells is enriched for both ζWT and positive control c-myc mRNAs (Figure 7C), demonstrating the assembly of ζ-globin/AUF1 mRNPs *in vivo*. These results document the interaction of AUF1 with the ZMRE and provide an attractive mechanistic link between the structure of the ζ-globin 3′UTR and its mRNA-stabilizing functions. The importance of AUF1 to this process also suggests that the half-life of ζ-globin mRNA may be regulated in definitive erythropoiesis through post-transcriptional programs that act on other mRNAs, including β-globin mRNA, that are central to normal red cell development.

## Discussion

Recent reports that ζ globin acts as a physiological surrogate for deficient α globin [8,9] and efficiently inhibits the pathological polymerization of deoxyHb S [7,10] have engendered substantial interest in developing this embryonic globin as a unique and highly effective therapeutic for α thalassemia and sickle-cell disease, respectively. The premise that developmentally silenced globin genes can be derepressed in definitive erythroid cells is consistent with observations that ζ globin is expressed at high levels in several congenital and acquired conditions in humans [24-26] and can be reactivated by mutations that target transcriptional regulatory elements in animals [22-24]. Developmental stage-discordant embryonic globins can also be expressed from transgenes that have been modified to contain adult-stage promoters and enhancers [6,17,22]. While the specific mechanisms that underlie transcriptional repression of embryonic globin genes in definitive erythropoiesis remain a matter of active investigation, it is clear that this process can be reversed in adult erythroid progenitors.

An equally important determinant of ζ-globin expression--its *post*-transcriptional regulation--has been studied less extensively in both primitive and definitive erythropoiesis. Processes that impart high stability to globin mRNAs are particularly important in adult erythroblasts, permitting these transcripts to accumulate to high levels and to translate globin protein for 3–5 days following nuclear condensation and extrusion from orthochromatophilic erythroblasts [1]. As might be predicted, mutations that impair the high stabilities of globin mRNAs in transcriptionally silent cells will disproportionately impact the levels of their encoded proteins. For example, a naturally occurring antitermination mutation that shortens the t_1/2_ of α-globin mRNA to ~25% of its normal value (α^Constant Spring^) coordinately reduces expression of the cognate globin monomer to ~2% of the wild-type value [49,50]. Without some knowledge of the half-life of ζ-globin mRNA, on the mechanism through which it is stabilized in definitive erythroid cells, it is difficult to predict whether transcriptional derepression of the ζ-globin gene transcription will necessarily achieve the desired therapeutic effect.

We previously observed a modest difference in the stabilities of human α-and ζ-globin mRNAs that were compelled to express in definitive mouse erythroid cells [18]. This effect, which mapped to single-nt differences in homologous pyrimidine-rich elements (PREs) positioned within the two 3′UTRs, was attributed to a 6-fold reduction in the affinity of the ζ-PRE for αCP (hnRNP E), a cytoplasmic mRNA-binding protein that effects the high stability of α-globin mRNA [12,18]. Importantly, ζ-globin mRNA was not fully stabilized by exchange of the ζ-PRE for the corresponding α-globin determinant, suggesting the activities of other, structurally dissimilar mRNA-stabilizing elements [18]. The present study validates this hypothesis, as it identifies a unique site-specific region of 3′UTR that is essential for the normal cytoplasmic accumulation of ζ-globin (Figures 1 and 2). Subsequent half-life analyses, conducted *in vivo* in intact cultured cells, directly demonstrate the importance of the ZMRE (and, specifically, its 4-nt cytosine-rich core) to the cytoplasmic stability of ζ-globin mRNA (Figures 3 and 4).

Our several measures suggest a t_1/2_ value of 11–15 hr for ζ-globin mRNA in HeLa cells (Figures 3 and 4), which is surprisingly close to a t_1/2_ value of ~11 hr for α-globin mRNA obtained in MEL cells using a similar strategy [50]. The similar stabilities of α- and ζ-globin mRNAs in these cultured cells--which do not express other globin mRNAs in significant quantities--contrasts sharply with their discordant stabilities in primary mouse cells that co-express high levels of endogenous α-globin mRNA [18]. The cell context-dependent difference in the relative stabilities of the α- and ζ-globin mRNAs suggests that they may be co-regulated through a shared post-transcriptional mechanism. We previously described a similar relationship among developmentally related β-like globin mRNAs, where the stabilities of transgenic embryonic ϵ- and fetal γ-globin mRNAs are reduced in the presence of adult β-globin mRNA [35,36]. A similar relationship between the ζ- and α-globin mRNAs would account for the significant reduction in the half-life of ζ-globin mRNA when co-expressed with α-globin mRNA of either human or mouse origin. The implications of this mechanism vis-à-vis α thalassemia are substantial, as expression of ζ-globin mRNA from a therapeutic transgene would self-correct to higher or lower levels in patients with more or less severe deficits in α-chain production, respectively.

Our results also indicate that the stability of ζ-globin mRNA in definitive erythropoiesis reflects a balance between at least two separate post-transcriptional programs. The ZMRE that we identify is distinct from the previously identified ζ-PRE in both its position within the 3′UTR as well as the specific *trans*-acting factors that it binds (Figure 7). Specifically, the ZMRE binds AUF1, a *trans*-acting mRNA-binding protein implicated in both the stabilization and destabilization of heterologous mRNAs in erythroid and non-erythroid cells [16,47,48]. This interaction may account for the observation that ZMRE-related activities are readily observed in non-erythroid HeLa cells (Figures 3 and 4), while αCP-mediated programs that stabilize α-globin mRNA are largely restricted to erythroid cells [11]. Importantly, AUF1 stabilizes β-globin mRNA through interaction with a 3′UTR determinant [16], suggesting potential mechanistic overlap between programs that direct the stabilities of α-like and β-like mRNAs or, conceivably, the stabilities of embryonic and adult globin mRNAs. Both possibilities would be consistent with dynamic post-transcriptional competition between embryonic and adult globin transcripts [36] and would predict that the efficiency of ζ-globin derepression in α thalassemia would be proportional to the severity of the α-chain deficit.

One remarkable property of the ZMRE is its apparent non-autonomous function (Figure 5), suggesting ‘allosteric’ effects of surrounding structures on the activity of this determinant (Figure 6). While our studies do not investigate the positions of these function-modifying structures, we think it unlikely that they reside in coding region mRNA which is constantly remodeled by actively translating ribosomes. It seems reasonable to speculate that ZMRE activity is instead enforced by interactions with other regions of 3′UTR (or factors that they bind), or by structural interface with the ζ-globin 5′UTR. The latter process might resemble the ‘closed-loop’ model for mRNA translation that invokes structural interactions between the 5′UTR, 3′UTR, and polyadenylate tail [51-55]. The experimentally determined structure surrounding the ZMRE is fully consistent with this possibility (Figure 6), predicting its positioning on an internal loop and/or within a hydrophobic nucleotide stack, where effector *trans*-acting factors might bind and subsequently alter the regional structure to reveal (or conceal) co-determinants of mRNA stability.

## Conclusion

Collectively, the present studies address the relevance of post-transcriptional regulatory events to ζ-globin expression in definitive erythroid cells, by defining structures and implicating mechanisms of ζ-globin mRNA stability. Our functional analyses identify a previously unrecognized determinant of mRNA stability within the ζ-globin 3′UTR whose position can be narrowed to a 4-nt site-specific cytosine sequence. Coordinate structural studies recognize that this region maps to a functionally germane mRNA form that is targeted by *trans*-acting regulatory factors that have been previously been implicated in stabilizing both globin and non-globin mRNAs. This information provides a foundation for subsequent mechanistic studies that will be critical for understanding the full utility of developmental stage-discordant ζ-globin mRNA as a therapeutic for important disorders of globin gene expression.

## Methods

### Gene construction

Parental pTRE2Aζ was derived from pTRE2 (Clontech) by inserting the full-length human ζ-globin gene (including ~100 nt of contiguous 5′- and 3′-flanking regions) into the polylinker *Sac*II-*Bam*HI site. A ~1.0 kb *Nco*I-*Sap*I vector fragment was subsequently deleted to eliminate a default polyadenylation signal that competes with the native ζ-globin poly(A) signal. 3′UTR-derivative ζ-globin genes were constructed from pTRE2Aζ by exchanging the exon III *Bst*EII-polylinker *Bam*HI fragment with 330-bp synthetic DNAs (Genscript), each encompassing a mutated ζ-globin 3′UTR. The construction of a corresponding full-length β-globin gene (pTRE2Aβ) is described elsewhere [15]. Genes encoding chimeric βζ-globin mRNAs were generated from pTRE2Aβ by exchanging the parental *Eco*RI-*Eco*NI DNA fragment (encompassing β-globin exon III and contiguous 3′-flanking region) for a corresponding synthetic DNA containing the desired wild-type or derivative ζ-globin 3′UTR. All recombinant DNAs were validated by automated sequencing.

### Cell culture

HeLa^tTA^ cells expressing the tetracycline transactivator fusion protein (tTA; Clontech) were maintained in DMEM supplemented with 10% FBS and antibiotics, at 37°C in a humidified 5% CO_2_ environment. Wild-type and 3′UTR-derivative pTRE2Aζ vectors used for stable transfections were modified by inserting a 1.5-kb DNA fragment encoding hygromycin (hyg) resistance into a unique vector *Xho*I site. Approximately 6×10^5^ cells were transfected with 5.0 μg of DNA using Superfect reagent under conditions recommended by the manufacturer (Qiagen), and selected with 400 μg/mL hyg. Hyg-resistant clones were tested by RT-qPCR for levels of ζ-globin mRNA and control endogenous β-actin mRNA. Tetracycline (tet) response was assessed by quantifying the level of ζ-globin mRNA, relative to the level of tet-indifferent β-actin mRNA, following a 48-hr incubation in tet-supplemented media (2 μg/mL).

### mRNA decay analyses

*Two-point decay analyses* were conducted on 5×10^5^ preplated HeLa^tTA^ cells maintained in tet-supplemented medium (0.5 μg/mL). Cells were transfected with 5.0 μg DNA comprising equal quantities of wild-type (or 3′UTR-derivative) TRE2Aζ and control pTRE2Aβ using Superfect reagent, and replated as two aliquots for overnight growth in tet-supplemented medium (0.03 μg/mL). PBS-washed aliquots were then incubated for five hr in tet-free medium; one aliquot was sacrificed immediately (t = 0) and a second aliquot after an additional 16-hr incubation in tet-supplemented medium (2.0 μg/mL). *Conventional half-life analyses* were conducted on 2×10^6^ HeLa^tTA^ cells maintained in tet-supplemented medium (0.5 μg/mL), transfected with 10 μg DNA using Superfect reagent, and then replated in aliquots in tet-supplemented medium (0.03 μg/mL) for overnight growth. PBS-washed aliquots were then incubated in tet-free medium for five hr, supplemented with tet (2.0 μg/mL) and sacrificed at defined intervals. For both transient and stable analyses, cells were sacrificed by immersion in Trizol, and RNA prepared as recommended by the manufacturer (Invitrogen). Purified RNA was resuspended at 10 μg/mL in H_2_O, and stored at −80°C.

### RT-qPCR

Purified RNA (50 ng) was assayed using Taqman One-Step reagents on a model 7500 real-time PCR system, using protocols recommended by the manufacturer (Applied Biosystems; AB). Analyses were conducted using assays for human ζ globin (AB catalogue HS00923579_m1), β globin (HS00747223_g1), and β actin (HS99999903_m1), and quantified by ΔΔCt methodology that we describe elsewhere [34]. Relative decay values were calculated as the quantity of each ζ-globin mRNA remaining after a 16-hr transcriptional chase interval, relative to transiently co-expressed β-globin mRNA; the relative decay of ζWT mRNA was arbitrarily assigned unit value. p values--where indicated--were calculated using standard Student t-test methods.

### Affinity enrichment

Custom 5′-terminal biotinylated single-strand (ss) DNAs corresponding to 3′UTRs from ζ^WT^ (5′TGGAGGTTCCCCAGCCCCACTTACCGCGTAAT3′), ζ^57^ (5′TGGAG GTTAGTGCACACCACTTACCGCGTAAT3′), and ζ^65^ (5′TGGAGGTTCCCCAGCCAGTGCACACGCGTAAT3′) mRNAs were commercially sourced (IDT). Approximately 1 μg of each ssDNA was incubated overnight at 4°C in 500 μL cytoplasmic extract supplemented with 50 μL ImmunoPure Avidin Agarose beads (Pierce). The pelleted beads were washed 2× with PBS + Triton-×100 (0.05%) and 2× with PBS + Triton-×100 (1.0%), resuspended in 10 μL loading buffer, and resolved on a precast 4-12% gradient SDS-polyacrylamide gel (Invitrogen). Cytoplasmic extracts were prepared from ~1×10^7^ cells lysed in buffer (1 mM Hepes pH 7.9, 0.15 mM MgCl_2_, 1 mM KCl) and clarified by centrifugation at 13000xg for five minutes at 4°C; extract was stored in aliquots at −80°C.

### Proteomics

Analyses were conducted by the University of Pennsylvania Proteomics Core Facility. Tryptic digests were studied by nanoLC/MS/MS using Thermo LTQ and Eksigebt nano LC-2 Da instruments, and data analyzed from the Uniprot_Sprot database using Sequest and Scaffold software packages. Statistical p-value cutoffs of 95.0% and 99.0% were applied for peptides and proteins, respectively.

### Western transfer

Antibodies were used at the following dilutions: AUF1 (kind gift of G Dreyfuss; 1:2000), replication protein A (Santa Cruz SC-81373; 1:300), and hnRNP-H (Santa Cruz SC-10042; 1:500). Cytoplasmic extracts were resolved on precast 4-12% SDS-polyacrylamide gels, then transferred to nitrocellulose using an XCell II Module *per* manufacturer recommendations (Invitrogen). Membranes were blocked for 30 minutes at room temperature (RT) with Superblock T20 (Thermo Scientific), then supplemented with primary antibody for 60 min and with secondary HRP-conjugated antibody for an additional 30 minutes. Immunoblots were washed thrice for five minutes at RT with PBS + Tween-20 (0.1%), and analyzed by ECL chemiluminescence (GE Healthcare).

### mRNA secondary structure

A DNA template for *in vitro* transcription of the ζ^WT^ 3′UTR, containing an 18-nt polyadenylate tail, was directionally inserted into the *Xho*I-*Bgl*II polylinker site of pSP72. The *Bgl*II-linearized plasmid was transcribed *in vitro* as previously described [41], and the purified RNA then 5′-end labeled with [γ-^32^P]ATP using a Kinase Max kit (Ambion). The [^32^P]-labeled RNA was digested with RNase A (100, 10, 1.0, and 0.1 ng/mL), RNase T1 (100, 10, 1.0, and 0.1 mU/μL), or RNase V1 (10, 1.0, 0.1, and 0.01 mU/μL) as directed by the supplier (Ambion). RNases A and T1 exhibit cleavage specificities for pyrimidine and guanosine bases in single-stranded regions of RNA, respectively, while RNase V1 cleaves 3′ to nucleotides within double-stranded regions of RNA. Reaction products were resolved on a 33 cm × 40 cm 6%:8 M acrylamide:urea gel, in parallel with a migration-control ‘ladder’ generated by alkaline hydrolysis of [^32^P]-labeled transcripts [41].

## Competing interests

The authors declare that they have no competing interests.

## Authors’ contributions

Participated in research design: ZH, DS, SvZ, JER. Conducted experiments: ZH, DS, SvZ. Performed data analysis: ZH, JER. Wrote or contributed to the writing of the manuscript: JER. All authors read and approved the final manuscript.
